# Extracorporeal shockwave therapy combined with alginate dressing for treatment of sacroiliac decubital necrosis in older adults: A case report

**DOI:** 10.1097/MD.0000000000019849

**Published:** 2020-05-08

**Authors:** Haoyang Duan, He Li, Hongying Liu, Hong Zhang, Na Liu, Qingjun Dong, Zhenlan Li

**Affiliations:** Department of Rehabilitation Medicine, The First Hospital of Jilin University, Changchun, Jilin, China.

**Keywords:** alginate, sacroiliac decubital necrosis, shockwave, therapy

## Abstract

**Rationale:**

Pressure sores are major clinical problems with limited treatment options.

**Patient concern:**

A 62-year-old man, admitted with unresponsive, indifferent mood, and malnutrition. A noval-shaped skin lesion deeping into the fascia was found in the sacrococcygeal region.

**Diagnosis:**

The patient was diagnosed with delayed carbon monoxide poisoning encephalopathy and stage IV sacrococcygeal pressure sores.

**Intervention:**

The patient received neurologic rehabilitation therapy and simple debridement dressing care followed by extracorporeal shockwave therapy.

**Outcomes:**

The scores for the pressure ulcer healing scale were 17 points, 13 points, 9 points, and 5 points, respectively, before treatment and at the 4-, 8-, and 12-week follow-ups. At 2 weeks after discharging the total pressure ulcer healing scale score was 0, which signifies that the sacroiliac decubital necrosis healed without adverse reactions and side effects.

**Lessons:**

This study presents a therapeutic solution for treating older adults with pressure ulcers by using extracorporeal shockwave with alginate dressing, which proved effective and safe.

## 1 Introduction

Pressure sores have been a major clinical problem in the recent years, due to limited treatment options and costly health care.^[1]^ As the aging population is increasing in China, there is the number of patients suffering from pressure ulcers. Since older patients suffer from various additional medical conditions, the healing time of pressure ulcers is slower, which not only affects recovery to health, but can also lead to infection and even death.^[2]^ Accelerating the healing rate of pressure ulcers can improve the patients’ quality of life and reduce the economic burden of the disease.^[3]^ The Department of Rehabilitation from the First Hospital of Jilin University admitted an older patient with carbon monoxide poisoning encephalopathy and sacroiliac decubital necrosis, and his wound was treated by extracorporeal shockwave therapy combined with alginate dressing, as described in this study.

## 2 Case report

### 2.1 Patient

A 62-year-old Korean male was hospitalized for treatment of cognitive disorder with decubital necrosis at the Department of Rehabilitation Medicine of the First Hospital of Jilin University, Changchun, China. The patient had history of carbon monoxide poisoning without any medical treatment for 40 days, and had hypertension, denied family history of genetic disease, epilepsy, and mental illness. One month ago, family members have found the patient unresponsive, gradually aggravating indifferent mood. Two weeks ago, the patient was found sacroiliac decubital necrosis by his family, which had not been treated effectively.

At admission, the patient had blood pressure of 140/100 mm Hg and was unresponsive, with indifferent mood and malnutrition. A noval-shaped skin lesion deeping into the fascia was found in the sacrococcygeal region. The approximately 7.5 cm × 5.5 cm area of necrotic tissue with purulent secretions was surrounded by red, swollen tissue. Blood tests showed white blood cell count 9.07 × 10^9^/L, red blood cell count 5 × 10^9^/L, platelet count 350 × 10^9^/L, hemoglobin 127 g/L, total protein 65.7 g/L, and albumin 37.9 g/L. The patient was diagnosed with delayed carbon monoxide poisoning encephalopathy and stage IV sacrococcygeal pressure sores.

### 2.2 Institutional review board statement

This research did not increase the risk and economic burden of the patient; the patients’ rights were fully protected; the project design was conducted in line with scientific and ethical principles. The institutional of the First Hospital of Jilin University review board approved this project.

### 2.3 Informed consent statement

The individual in this study has provided informed written consent to publish the case details.

### 2.4 Treatment and measurements

#### 2.4.1 Treatment

Following admission, the patient received neurologic rehabilitation therapy, for 1.5 hours per session, twice daily, 6 days a week, for 12 weeks. The necrotic area was treated by simple debridement dressing (once every 2 days), followed by extracorporeal shockwave therapy (Swiss Dolorclast Company, Nyon, Switzerland) once a week, for 12 weeks. The parameters for shockwave therapy were as follows: pressure 2 to 3 bar, frequency 4 to 5 Hz, pulse number 200/300 + 100/cm^2^. After informing the patient and family of the basic process and possible adverse reactions, the patient was seated in a prone position, with completely exposed pressure ulcers. The wound was thoroughly cleaned of necrotic tissue and covered with double sterile gauze, followed by sterile plastic film evenly coated with coupling agent. Shockwave probes of 15 mm were placed in the center of the wound and moved at a constant speed in circular motion, with a radius gradually increasing from the center to the rim. After the shockwave therapy, the skin surrounding the wound was disinfected with 5% iodine and the wound was cleaned with isotonic saline and covered with an alginate dressing block (5 × 5 cm^2^ Urgosorb; Urgo, Paris, France) of a size appropriate to the wound depth and surface. The outermost layer was vaseline-oil moisturizing gauze covered with a transparent sticker. Considering the patient with cognitive disorder followed by the delayed carbon monoxide poisoning encephalopathy, he was received the cognitive therapy treatment once a day, 6 times a week, for 12 weeks.

**Figure 1:**
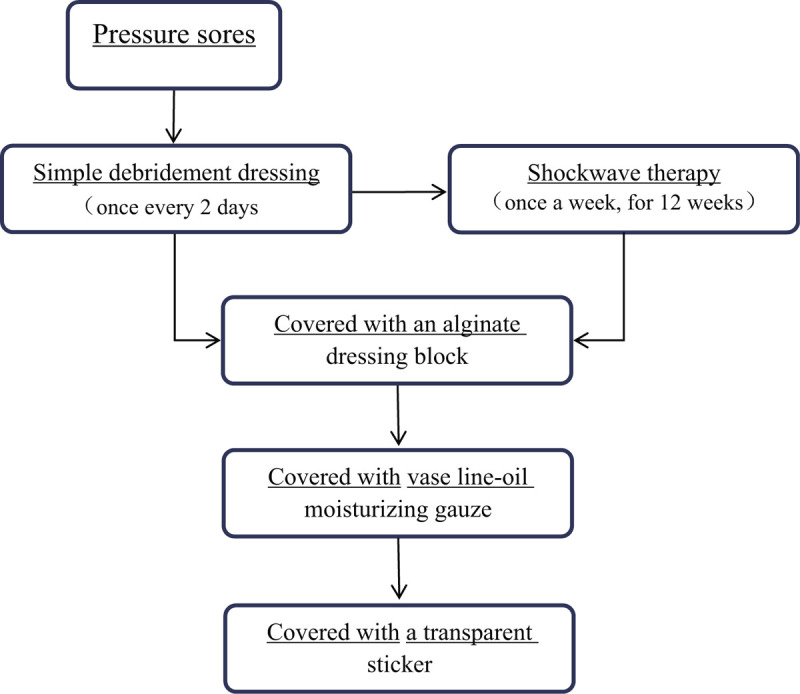
The schedules of treatment received by the patient.

The schedules of treatment for the patient received are shown in Figure 1.

#### 2.4.2 Measurements

The pressure ulcer healing scale (PUSH)^[4]^ was used to evaluate the ulcer before treatment and at the 4-, 8-, and 12-week follow-ups. The following parameters were used to assess the wound: surface area from 0 to 24 cm^2^, assigned from 0 to 10 points; exudate evaluated as nothing, small, medium, and large amount and assigned 0 to 3 points, respectively; and type of wound tissue divided into closed, epithelial tissue, granulation tissue, carrion, and necrotic tissue and assigned 0 to 4 points. The final score ranged from 0 to 17 points, with lower scores being indicative of condition of the pressure sores. The treatment was deemed as cure if the PUSH score was 0 points, as effective if the total score decreased by more than 3 points, or as ineffective if the total score decreased by <3 points. The Rancho Levels of Cognitive Functioning was assessed the patient's cognitive function, before treatment and at the 4-, 8-, and 12-week follow-ups.

## 3 Results

**Figure 2:**
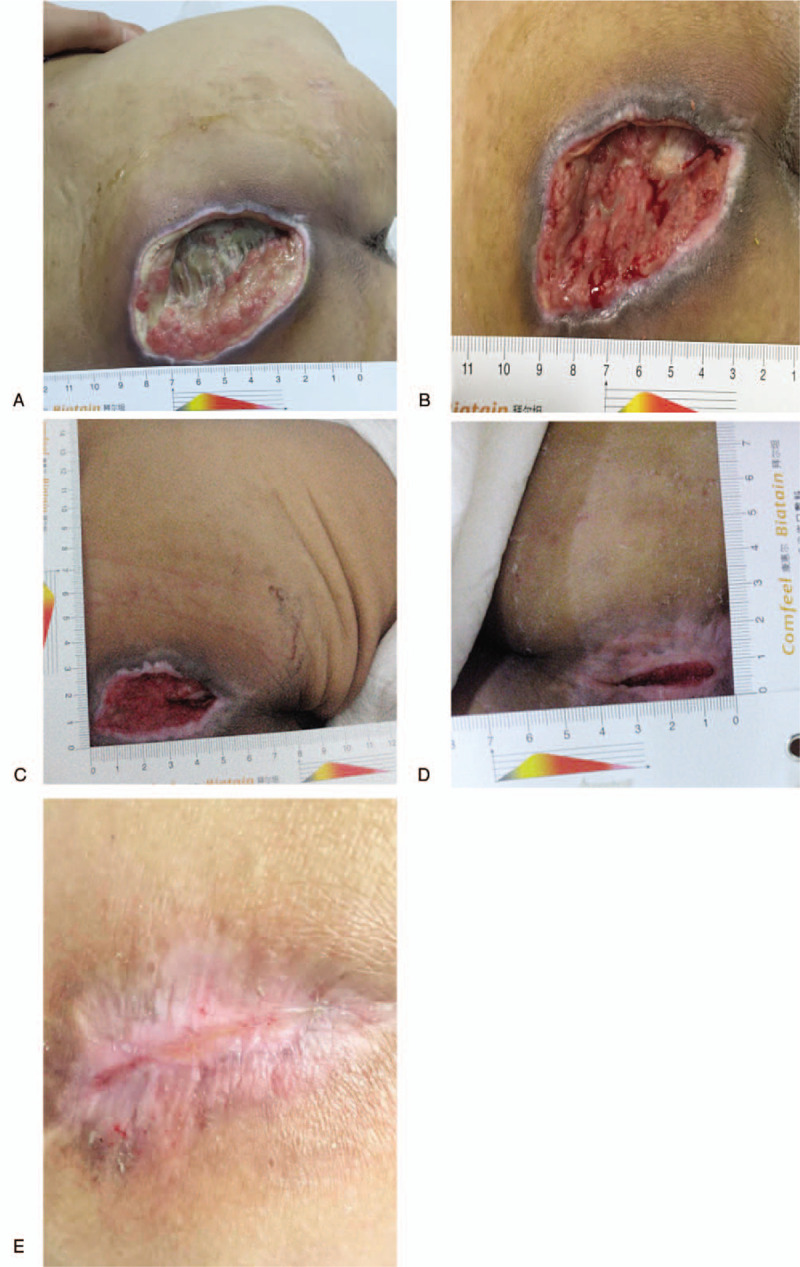
(A) Before treatment. (B) Four weeks after therapy. (C) Eight weeks after therapy. (D) Twelve weeks after therapy. (E) Two weeks after discharge.

The PUSH index scores for surface area, exudate, and wound tissue before treatment were 10, 3, and 4 points, respectively, for a total score of 17 points (Fig. 2 A). After 4 weeks of treatment, the PUSH index score dropped to 9 points for surface area, 2 points for exudate, and 2 points for wound tissue, with a total score of 13 points. Since the total score decreased by 4 points, the evaluation was deemed as effective (Fig. 2B). After 8 weeks, the PUSH index score for each parameter was 7, 0, and 2 points, respectively, with a total score of 9 points, deeming it an effective evaluation (Fig. 2C). At 12 weeks, each PUSH index score was 3, 0, and 2 points, respectively, with a total score of 5 points, down by 4 points from the previous evaluation, thus the treatment was effective (Fig. 2D). After 12 times of treatment, there were no adverse reactions and side effects. The patient was followed for another 2 weeks after discharging, at which time the total PUSH score dropped to 0 points, therefore the sacroiliac decubital necrosis was considered healed (Fig. 2E). Before cognitive rehabilitation treatment, the patient's cognitive function was Rancho level IV. After 4 weeks of cognitive rehabilitation, his function still showed Rancho level IV. But after 8 weeks of cognitive rehabilitation, his function improved to Rancho level V and after 12 weeks of cognitive rehabilitation, his function improved to Rancho level VI.

## 4 Discussion

The sacral tail area is a common region for pressure ulcers, because the soft tissue around the sacral tail is pachynsis, there are much easier to form fistula or pouch type of pressure ulcers, and expand quickly into the surrounding and deep tissue. When the pressure ulcers grow bigger, they accumulate large amounts of water, heat, and proteins, gradually leading to malnutrition, hypoalbuminemia, or sepsis, which can eventually result in death.^[5]^ Compared with younger adults, elderly patients have less subcutaneous fat and moisture, lower soft-tissue regeneration and repair ability, often a variety of underlying medical problems, and difficulty in communication, thus, making the treatment process more difficult.

The causative mechanism for pressure ulcers is currently still controversial. Most studies implicate long-term local stress which results in blood circulation dysfunction, limited oxygen supply, tissue damage, and eventually necrosis.^[6]^ It is of outmost importance to prevent the occurrence of pressure ulcers; however, once they occur, the main treatment includes local wound dressings and local pressure alleviation. Due to their inefficiency for large ulcers, treatments such as topical negative pressure drainage, growth factors therapy, and hyperbaric oxygen have been introduced for testing, but they still lack high-level medical evidence.

Extracorporeal shockwave is a new method used in the treatment of diabetic foot ulcers and soft tissue wound healing, but there are only few reports^[7]^ about the treatment of pressure ulcers. There are a few proposed mechanisms underlying the treatment of pressure ulcers. One is through inhibition of inflammation, since low-energy extracorporeal shockwaves can modulate the quantity of nitric oxide, improve the activity of endothelial nitric oxide synthase in cells, effectively reduce the expression of proinflammatory cytokines, and overall attenuate the inflammatory reaction.^[8]^ Another proposed mechanism is through an increase in the permeability of bacterial membranes, thus enhancing the sensitivity of bacterial peptidoglycan to destruction and supporting the process of sterilization and bacteriostasis.^[9]^ Also, extracorporeal shockwaves can improve the local blood supply, by promoting angiogenesis and tissue blood per fusion. This therapeutic approach can also activate mesenchymal stem cells, which secrete a variety of cytokines responsible for wound angiogenesis, necessary for repair and healing.^[10]^ The last proposed mechanism is through an increase in tissue regeneration. Extracorporeal shockwaves can increase the local blood flow perfusion, speed up wound re-epithelization, promoting accelerated wound healing.^[11]^

Alginate dressings are made of alginate, a natural polysaccharide carbohydrates extracted from seaweed. The material has high absorbency, and when it comes in contact with wound exudates, it becomes a soft gel, which provides the wound with a wet environment, necessary for wound healing. In addition, this dressing can alleviate pain, due to a continuous and stable antibacterial effect. It can be used for medium to severe seepage, infection, or pressure ulcers with risk of infection.^[12,13]^

Based on the health condition of the patient, pressure ulcers are addressed by debridement and wound dressing every 2 days and removal of the necrotic tissue, to stabilize the patients’ vital signs and to prevent infection. Before extracorporeal shockwave therapy, the nurse must thoroughly clean the necrotic tissue, and after extracorporeal shockwave therapy, alginate dressings must be used to fill the pressure ulcers. After 12 weeks of combination therapy and 2 weeks of follow-up, our patient's large pressure ulcers were fully healed, demonstrating that the extracorporeal shockwave can change the physical environment of the wound, stimulate local blood vessel growth, accelerate the release of active substances, and promote the growth of granulation tissue and wound healing. The alginate dressing absorbs the drain fluids by forming a gel, which provides creates a slightly acidic environment, maintains the active substance in the drainage, and promotes the formation of granulation tissue.

We applied alginate dressings to this patient according to the instructions. It is worth noting that alginate dressings cannot be used for wounds with less exudate and wounds covered with dead-tissue crusts, especially 3rd-degree burns with exposed tendons or cartilage. But alginate dressings are highly absorbent and anti-infective, and they are very suitable for more oozing infected wounds, such as more oozing infected pressure sore wounds.^[14]^ Currently, alginate dressings can be used in combination with other dressings, such as hydrocolloid dressings, foam dressings and charcoal (control odor), and silver ion dressings for different wound treatments. A few research have been discussed when the tendon or cartilages exposed to alginate therapy. Sayag et al^[15]^ compared the efficacy of an alginate (Algosteril) with that of dextranomer paste, an established local treatment, in a prospective, randomized, controlled trial of 92 patients with pressure ulcers of grade III or IV. The authors concluded that the striking efficacy of the alginate dressings suggested that they may have pharmacologic properties to wound healing.

According to the caregiver's description, the patient showed indifferent mood in the early stage after attacked by carbon monoxide poisoning encephalopathy. He did not like to communicate with families, even not to like move and active, lay in bed at unchangable position all the days. One month later his family found his sacrococcygeal pressure sores, while the patient presented to the cognitive disorder, especially insensitive to any pain and body position which deteriorate the serious of pressure ulcers.

After admitted to our department the patient received cognitive rehabilitation and showed significant improvement of cognitive with mood changes. When he was assessing his cognitive function, the scale was cognitive level IV, which showed indifferent mood, that he could not respond to environment around him, difficulty following directions and be very confused and frightened. After 8 weeks of cognitive rehabilitation treatment, the patient's cognitive function improved to cognitive level V, which presented to be able to pay attention for a few minutes, communicate with his family a few word, try to act followed simple instructions, remember a few past events, and recognize family members. After 12 weeks of cognitive rehabilitation treatment, the patient's cognitive function improved to cognitive level VI, which could like to talk with his family, follow some instruction with assistance, willingness to perform simple action and could be able to do most self-care ability of activities of daily living (ADL). He was sensitive to external stimuli, especially pain when he received the shock wave treatment. With the improvement of cognitive function and mood change, the patient was able to start to complete everyday activities following instruction and gradually reduce the time lying in bed, thus reduce the pressure of sores. From this patient's clinical observation, we considered that cognitive rehabilitation treatment might promote the patient's mood change and sensation, which could help him to prevent and alleviate the deterioration of the pressure ulcers.

Wurzer et al^[16]^ consider there was a risk of recurrence of pressure ulcers for the patient especially with the large area of pressure ulcers. In this case, the patient with relatively large area of pressure ulcers might be a higher recurrence, therefore the patient and his caregivers were advised some prevention strategies referred by the Wound, Ostomy and Continence Nurses Society (WOCN) according to the Guideline Prevention and Management of Pressure Injuries (Ulcers) An Executive Summary as following. Firstly, pay more attention to observe the compression of the skin, and give proper massage. Secondly, necessary to change the body position frequently and use the special mattress to ease pressure in sacrum caudal region. Finally, by giving the patient a high-protein and high-vitamin diet, improving the nutritional status of the body and ensuring positive nitrogen balance, enhancing the body resistance and repair ability.^[17]^

This case presented a new therapeutic approach for pressure ulcers in older adults by using extracorporeal shockwave therapy with alginate dressing. Certainly the cognitive rehabilitation might be helpful for the patient's recovery from health and function, but it is considered that extracorporeal shockwave therapy combined with alginate dressing might be a new physical therapy which was effective and safe. It needs further research for extracorporeal shockwave therapy about the treatment scheme, the optimization and the specific underlying mechanism of it.

## Author contributions

Zhenlan Li conceived and designed the therapeutic methods.

Haoyang Duan performed the treatment and wrote the manuscript.

He Li helped perform the treatment and contributed to manuscript preparation.

Hongying Liu helped Perform the treatment.

Hong Zhang helped perform the analysis with constructive discussions.

Na Liu record and analyzed the data.

Qingjun Dong helped analyze the data.
